# Pregnancy outcomes after exposure to crisis pregnancy centers among an abortion-seeking sample recruited online

**DOI:** 10.1371/journal.pone.0255152

**Published:** 2021-07-28

**Authors:** Alice F. Cartwright, Katherine Tumlinson, Ushma D. Upadhyay

**Affiliations:** 1 Department of Maternal and Child Health, Gillings School of Global Public Health, University of North Carolina at Chapel Hill, Chapel Hill, NC, United States of America; 2 Carolina Population Center, University of North Carolina at Chapel Hill, Chapel Hill, NC, United States of America; 3 Department of Obstetrics, Gynecology & Reproductive Sciences, Advancing New Standards in Reproductive Health, University of California, San Francisco, Oakland, CA, United States of America; Western Oregon University, UNITED STATES

## Abstract

**Introduction:**

More than 2,500 crisis pregnancy centers (CPCs), which seek to convince people considering abortion to continue their pregnancies, exist in the United States. However, the characteristics of people who visit CPCs and their pregnancy outcomes are largely unknown. This study sought to describe the characteristics of people considering abortion who report visiting CPCs, and whether CPC visit is associated with abortion or continuing the pregnancy 4 weeks later.

**Methods:**

Between August 2017 to May 2018, we recruited pregnant people searching for abortion services online, and 857 participants completed baseline and 4-week follow-up surveys. We described characteristics associated with visiting a CPC and compared pregnancy and abortion outcomes for those who reported CPC visit to those who did not using mixed-effects multivariable logistic regression.

**Results:**

Overall, 13.1% of respondents visited a confirmed CPC. Living further away from a CPC was associated with lower odds of a CPC visit. At follow-up, respondents who had visited a CPC were significantly less likely to have had an abortion (29.5%) than those who had not visited a CPC (50.5%). In the adjusted models, respondents who had visited a CPC had higher odds of being pregnant and still seeking abortion (aOR: 2.26, 95% CI: 1.37–3.73) or continuing the pregnancy (aOR: 2.35, 95% CI: 1.33–4.15) (versus having had an abortion), than those who had not visited a CPC.

**Conclusions:**

CPCs may be providing resources to people who are considering continuing their pregnancy and/or they may be misleading people about the care and referrals they provide related to abortion. Pregnant people need access to accurate information, decision support, and resources to make the pregnancy or abortion decision that is best for them.

## Introduction

Pregnancy resource centers, or crisis pregnancy centers (CPCs), are generally religiously-affiliated non-profits with physical locations that operate with the goal of convincing pregnant people considering abortion to parent or place their child for adoption and not to have abortions [1, 2]. These centers often provide pregnancy tests, free ultrasounds, and other material support to pregnant people and have proliferated over the past few decades, with more than 2,500 CPCs recently documented in the United States (U.S.), compared to fewer than 800 abortion facilities [3, 4]. CPCs in over 30 states receive state funding through multiple mechanisms [5–7] and recent changes in federal policy allow CPCs to be awarded funds through the Title X family planning program [8, 9].

Even as CPCs increase, the number and characteristics of the people who visit CPCs is less well known. While there are an estimated 2.8 million unintended pregnancies annually in the U.S. [10], it is difficult to generate an accurate estimate of how many people visit CPCs. eKYROS, a client management software platform used by CPCs, documented 361,449 new client visits to CPCs in 2019 [11]. While the data quality presented on eKYROS’ website and its true coverage of CPC visits is unknown, they claim to be utilized by both Care Net and Heartbeat International, two large evangelical organizations that support CPC affiliates in the U.S. One of the few peer-reviewed studies that attempted to quantitatively describe who visits CPCs in Southern Louisiana found respondents seeking prenatal and abortion care were equally likely to have visited a CPC (5% of prenatal and 6% of abortion patients) [12]. They also determined that older age was significantly associated with a CPC visit, whereas there were not significant differences by race/ethnicity or number of previous pregnancies between those who visited a CPC and those who did not.

Since some abortion patients in the Louisiana study confirmed visiting a CPC for their current pregnancy, there were evidently some people for whom the CPC did not have its intended effect of preventing abortion. In addition, qualitative interviews with prenatal care patients from the same Louisiana study found that over three-quarters of those who had visited a CPC were already planning to continue their pregnancy and parent when they visited a CPC and were seeking pregnancy tests, emotional support, or material goods [12]. Those who reported considering abortion and then continued their pregnancy cited other factors contributing to their decision, including family opposition to abortion, uncertainty about the abortion decision, and difficulty accessing an abortion facility.

Pregnant people considering abortion continue to face challenges accessing abortion care and CPCs often present themselves as locations to discuss “pregnancy options”. State abortion restrictions have led to a reduction in the number of abortion clinics in many states [13, 14]. The need to travel to obtain abortion care falls disproportionately on Black people, those with less education, and those who have to pay out-of-pocket for care [15]. The lack of access to abortion facilities may result in people with less resources and those who live in areas with fewer abortion facilities being more likely to seek pregnancy or abortion-related care from CPCs.

There continues to be limited information in the reproductive health literature about the role of CPCs among pregnant people considering abortion. The purpose of this study is to describe characteristics of those visiting CPCs drawn from a national sample of pregnant people searching for abortion information and services online and explore the association of CPC exposure with pregnancy and abortion outcomes.

## Methods

Data come from the Google Ads Abortion Access Study, a prospective study among pregnant people in the U.S. searching online for and considering abortion. The primary objective of the main study was to understand how structural, interpersonal, and individual factors impact the likelihood of obtaining a wanted abortion. The detailed methods of the study are published elsewhere [16], but in brief, data were collected from August 2017 to May 2018. Recruitment into the study was through advertisements appearing in Google search results, displayed to individuals using abortion-related search terms. We recruited a state-stratified sample, with ads displayed in all 50 states and the District of Columbia, with the initial goal of reaching a sample of at least 20 respondents per state. The number of responses per state was monitored to ensure that the sample was not disproportionately from only the largest states by population; in 8 smaller states, we ultimately recruited less than 20 participants [16].

Respondents were eligible for participation if they responded affirmatively to being currently pregnant and considering abortion. They provided electronic informed consent, completed a short baseline survey online, and provided an email address and phone number to be contacted for follow-up. The baseline survey asked participants to provide the date of their last menstrual period, the nature of their relationship with the person they became pregnant with, who gets to make the decision about what happens to the pregnancy, and their number of previous pregnancies, live births, abortions, and miscarriages. Participants were also asked to complete the Decision Conflict Scale (DCS), a 16-item scale previously used to assess conflict about health care decisions, including abortion [17, 18], and questions from two validated scales of abortion stigma [19, 20]. Finally, respondents provided their age, self-identified race/ethnicity, highest level of completed education, including whether they were currently a student, employment status, whether they had enough money to meet their basic needs in the past 12 months, current marital and relationship status, health insurance coverage, religion and religiosity, and current city, state, and zip code of residence.

Four weeks later, participants received an email or text message invitation to complete the follow-up survey online. The follow-up survey asked participants what had happened with their pregnancy since the baseline survey, what factors made it harder to get an abortion, the DCS scale, how easy or difficult it was to decide whether to have an abortion, whether they had visited a CPC, and whether they had attempted to self-manage their abortion. In addition, those who were still pregnant at follow-up were asked what steps, if any, they had taken to get an abortion and whether at this point, they wish they had had an abortion. Those who had had an abortion at follow-up were asked what factors made it easier for them to get an abortion, the date and location of their abortion, and financial costs associated with the abortion.

All eligible participants who completed the follow-up survey were remunerated with a $50 gift card. Those who reported experiencing a miscarriage, live birth, or “other” pregnancy outcome were not eligible to complete the follow-up survey on their experience trying to access abortion. The study was approved by the Institutional Review Board at the University of California, San Francisco.

### Data

The primary outcome of interest was pregnancy or abortion outcome at the 4-week follow-up, categorized as: 1) had an abortion; 2) pregnant and still seeking an abortion; and 3) pregnant and planning to continue the pregnancy.

**Fig 1:**
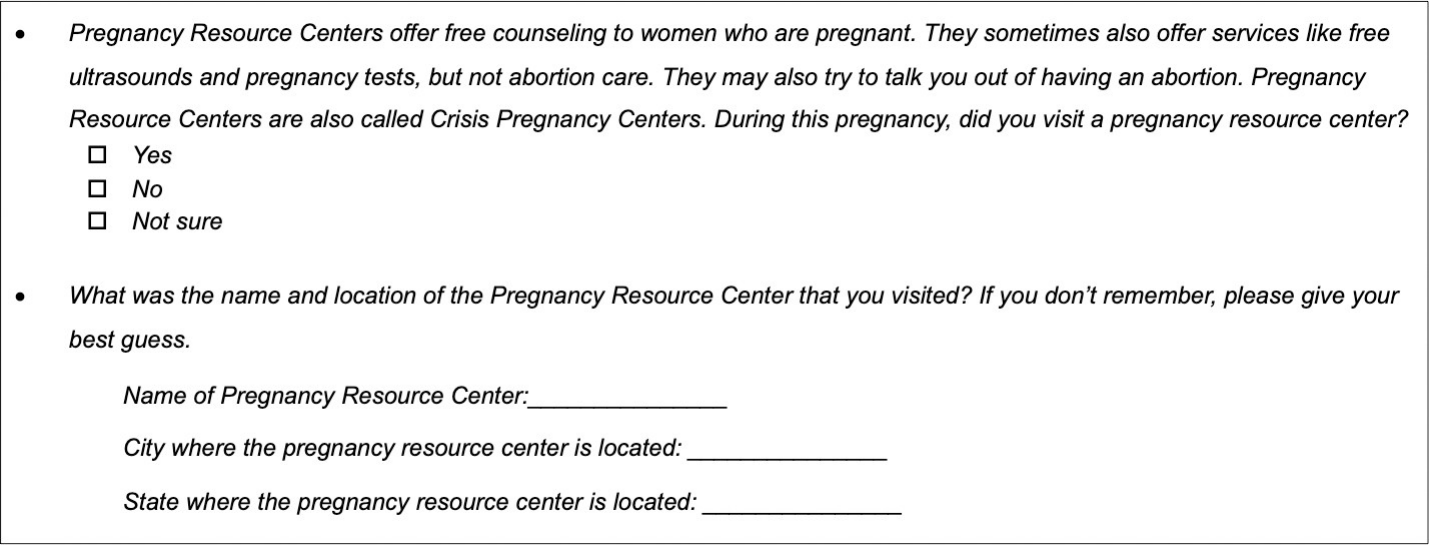
CPC questions in Google Ads Abortion Access Study 4-week follow-up survey.

The main independent variable of interest was exposure to a CPC. In the follow-up survey, participants were asked whether they visited a CPC (Fig 1). Participants who answered either “yes” or “not sure” were asked to provide the name of the CPC, as well as the city and state in which it was located. A research assistant reviewed all the CPC names and locations using Google searches to verify the responses. These were cross-checked by the first author using the Crisis Pregnancy Center Map and Finder [21] and additional Google searches. Respondents who answered “yes” to visiting a CPC but did not provide the name of the CPC, provided a general name such as “women’s clinic”, or otherwise provided a specific name that could not be verified were conservatively categorized as *not* having visited a CPC because the reported CPC could not be confirmed.

Key covariates provided by respondents in the baseline survey included their age, self-identified race/ethnicity, highest level of education completed, and geographic region of residence. Those who reported that they had had enough money to meet their basic living needs “some of the time”, “rarely”, or “never” were classified as having difficulty meeting their basic needs. Respondents also reported whether they currently had any type of health insurance, whether they belonged to a religion and which one, and how religious or spiritual they considered themselves. Baseline gestation was grouped into three categories: less than or equal to 10 weeks (gestation at which first-trimester medication abortion and procedural abortions are more widely available), 10.1–14.0 weeks (later first and early second trimester gestations, for which abortion may be more difficult to access and become more expensive), and greater than 14 weeks (gestations at which abortion are much less accessible in many states) [4]. Consistent with previous research, a transformed summary DCS score across the 16 scale items ranging from 0–100 was generated, with higher scores indicating higher decision conflict. Scores <25 are associated with success in implementing decisions, while scores >37.5 are associated with delayed decision making or uncertainty about implementing a decision [22].

Driving distance in miles from each participant to the nearest CPC and abortion facility was calculated in ArcGIS using the “Driving Distance” analysis function. The participant’s location was approximated using the longitude and latitude of the centroid of their zip code provided at baseline from the 2010 U.S. Census Zip Code Tabulation Areas [23]. The exact geographic location of the CPCs and abortion facilities was determined using their physical addresses. The sources of this information were the Crisis Pregnancy Center Map and Finder [21] and Advancing New Standards in Reproductive Health’s Abortion Facility Database containing data on all publicly advertised U.S. abortion facilities generated through a systematic online search in 2017 [4, 24].

**Fig 2:**
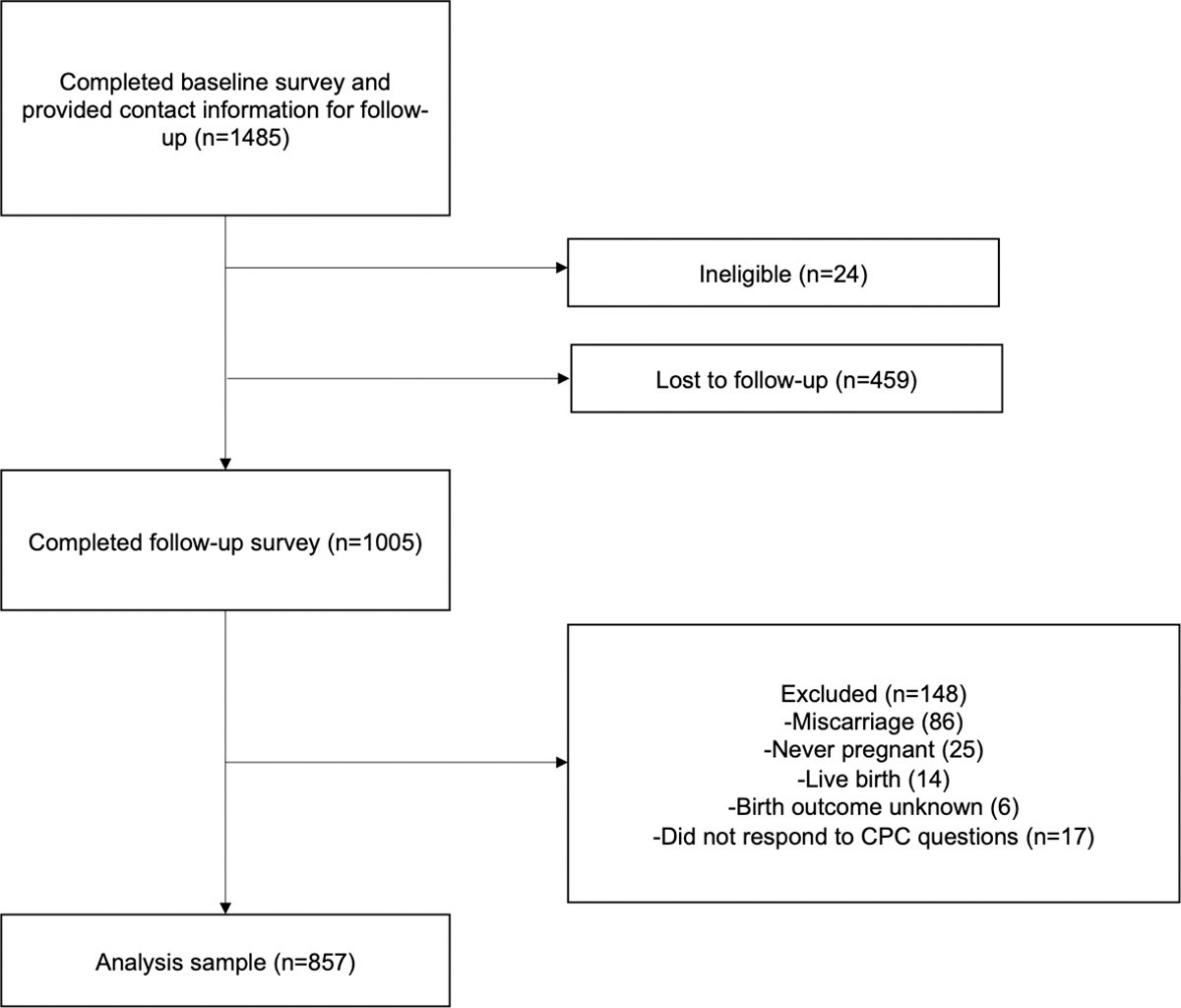
Loss to follow-up and reasons for exclusion in final sample.

Participants who had had an abortion or were still pregnant at follow-up and responded to the CPC questions constituted the final sample for this analysis. As described previously, a total of 1,485 people completed the baseline survey and provided contact information [16]. Among these, 24 were ineligible and excluded because they were either from outside the U.S. (n = 3) or attempted to complete the survey multiple times (n = 21). From baseline to follow-up, 459 participants were lost-to-follow-up (31%). Respondents who reported a non-eligible pregnancy outcome at follow-up (n = 131) and those who were missing responses to the CPC questions (n = 17) were also excluded, leaving a final sample of 857 (Fig 2).

### Analysis

For this analysis, we were interested in the association between exposure to a CPC and pregnancy or abortion outcome. First, we described the characteristics of the sample. We also examined whether there were sociodemographic differences between the final sample (n = 857) and those lost to follow-up (n = 459) using chi-square tests. We then used bivariate mixed effects logistic regression models to assess characteristics associated with visiting a confirmed CPC. We conducted a bivariate analysis of pregnancy outcomes by CPC exposure, comparing differences in outcome using chi-square tests. Finally, we conducted a mixed effects multinomial logistic regression to examine the relationship between visiting a confirmed CPC and pregnancy outcome. Having had an abortion at follow-up was the reference group for the multinomial model. The multivariable model adjusted for the sociodemographic, pregnancy, and residence characteristics described above that were hypothesized *a priori* to have a relationship with the exposure and outcome, and accounted for clustering at the state level. To examine the sensitivity of results to misclassification on exposure to CPCs, we re-ran the bivariate analyses and multivariable model re-classifying those who reported visiting a CPC but had missing or unverifiable CPC information as exposed (n = 50). Finally, as an additional sensitivity analysis in the multinomial model, we developed inverse probability weighted estimators (a form of propensity score analysis) to control for possible unmeasured confounding related to CPC visit. The propensity scores were estimated using a logistic regression model, with CPC visit as the dependent variable and factors hypothesized to be associated with CPC visit as the independent variables. After weighting, the standardized difference between the two groups was less than our threshold of 10% of the standard deviation of the outcome from the full sample. The mixed effects multinomial logistic regression model was then re-run, adjusting for the propensity scores. All analyses were done using Stata/SE 15.1. We considered a p-value of 0.05 or below to be statistically significant.

## Results

Of the 857 eligible respondents, 260 (30.3%) reported that they visited a CPC or were not sure. Of those who reported they had or may have visited a CPC, 63 of the 260 (24.2%) provided the name of an abortion provider, 35 (13.5%) provided the name of a medical provider (such as an obstetrician/gynecology practice, hospital, or federally qualified health center) and 50 (19.2%) did not provide a name or provided a non-specific name, such as “women’s clinic.” After review, we determined that 13.1% (n = 112) of respondents had visited a confirmed CPC.

Table 1 shows the demographic, pregnancy, and residence characteristics of the sample. A larger proportion of participants who had visited a confirmed CPC lived in the Midwest and less lived in the Northeast. In addition, a greater proportion of participants who had visited a CPC lived less than 5 miles from one, compared to those who had not been to a CPC. There were no differences between the groups in terms of distance to an abortion facility. Compared to the respondents who were lost to follow-up, respondents in this analysis were significantly more likely to be older than 24 years and to have private health insurance, and significantly less likely to be Black or Latinx, have a high school education or less, have difficulty meeting their basic needs, and to have had a previous birth.

**Table 1:** Sociodemographic, pregnancy, and residence characteristics of participants at baseline, by confirmed crisis pregnancy center visit.

Characteristic (%)	Visited a confirmed CPC	Did not visit a confirmed CPC	Total
	(n = 112)	(n = 745)	(n = 857)
Total	13.1	86.9	100
*Sociodemographics*			
**Age**			
<25	36.6	34.1	34.4
25–34	50.0	51.9	51.7
35+	13.4	14.0	13.9
**Race/Ethnicity**			
White	56.3	54.5	54.7
Black/African-American	19.6	25.0	24.3
Hispanic/Latinx	11.6	11.8	11.8
Asian, Multiracial, Other†	12.5	8.7	9.2
**Education**			
High school graduate or less	41.1	45.2	44.7
Some college, college or professional degree	58.9	54.8	55.3
**Has difficulty meeting basic needs**	48.2	45.8	46.1
**Health insurance**			
Private	18.7	25.8	24.8
Medicaid/Medicare/State exchange	53.6	51.9	52.2
None	25.9	19.6	20.4
Not sure/Other	1.8	2.7	2.6
**Religion**			
Catholic/Protestant	18.7	22.7	22.2
Other†	16.1	15.8	15.9
No religion/Don’t know	65.2	61.5	61.9
**Religiosity**			
Not at all religious/spiritual	26.8	29.3	28.9
Somewhat or very religious/spiritual	73.2	70.7	71.1
**Region**			
West	23.2	23.6	23.6
Midwest	35.7	24.4	25.9
Northeast	11.6	19.5	18.4
South	29.5	32.5	32.1
*Pregnancy history*			
**Gestation of pregnancy**			
Less than or equal to 10 weeks	75.0	79.7	79.1
10.1–14.0 weeks	15.2	11.4	11.9
14.1-weeks or greater	6.2	7.1	7.0
Missing	3.6	1.7	2.0
**Had previous abortion**	25.9	30.2	29.6
**Had previous birth**	65.2	66.2	66.0
*Decision conflict*			
**Baseline decision conflict score**			
Low conflict (<25)	47.3	53.3	52.5
Medium conflict (25–37.5)	19.6	21.6	21.4
High conflict (>37.5)	33.0	25.1	26.1
*Access to CPCs and abortion facilities*			
**Distance to nearest CPC**			
Less than 5 miles	57.1	44.4	46.1
5–24 miles	33.9	45.4	43.9
25+ miles	8.9	10.2	10.0
**Distance to nearest abortion facility**			
Less than 5 miles	25.0	27.5	27.2
5–24 miles	39.3	44.3	43.6
25+ miles	35.7	28.2	29.2

†Other race/ethnicity includes native Hawaiian, Pacific Islander, American Indian, and Alaska Native; Other religion includes Buddhism, Hinduism, Islam, Judaism, and Mormonism.

In the bivariate analysis looking at factors associated with visiting a confirmed CPC, distance from respondents’ home zip codes to CPCs was significantly associated with visiting a CPC, with further distance from CPCs associated with a reduced odds of visiting one (odds ratio (OR) = 0.57, 95% CI: 0.37–0.88) (Table 2). In the sensitivity analyses which included possible misclassified respondents, those who reported having Medicaid/Medicare health insurance (OR = 1.66, 95% CI: 1.04–2.65) or no health insurance (OR = 2.02, 95% CI: 1.18–3.46) (versus private insurance) had significantly higher odds of having visited a CPC. Additionally, baseline pregnancy gestation of 10.1–14.0 weeks compared to less than 10 weeks was significantly associated with greater odds of visiting a CPC (OR = 1.77, 95% CI: 1.08–2.91).

**Table 2:** Bivariate odds ratios for factors associated with exposure to a confirmed crisis pregnancy center (CPC) and exposure to a CPC with possible misclassification (N = 857).

Characteristic (reference group)	Confirmed CPC	CPC with possible misclassification
	OR (95% CI)	OR (95% CI)
**Age** (25–34)		
<25	1.11 (0.72–1.72)	1.09 (0.74–1.59)
35+	0.98 (0.53–1.81)	1.07 (0.63–1.79)
**Race/Ethnicity** (White)		
Black/African-American	0.76 (0.45–1.29)	0.92 (0.59–1.42)
Hispanic/Latinx	0.94 (0.49–1.81)	1.22 (0.71–2.08)
Asian, Multiracial, Other†	1.39 (0.73–2.64)	1.29 (0.72–2.32)
**Education** (Some college, college or professional degree)		
High school graduate or less	0.84 (0.56–1.26)	0.89 (0.62–1.26)
**Has difficulty meeting basic needs**	1.12 (0.75–1.67)	1.26 (0.89–1.79)
**Health insurance** (Private)		
Medicaid/Medicare/State exchange	1.44 (0.85–2.46)	1.66 (1.04–2.65)*
None	1.81 (0.99–3.34)	2.02 (1.18–3.46)**
**Religion** (None/Don’t know)		
Catholic/Protestant	0.78 (0.46–1.32)	1.24 (0.82–1.89)
Other†	0.95 (0.54–1.67)	1.14 (0.70–1.85)
**Religiosity** (Not at all religious/spiritual)		
Somewhat or very religious/spiritual	1.12 (0.71–1.76)	1.21 (0.82–1.79)
**Gestation of pregnancy** (Less than or equal to 10 weeks)		
10.1–14.0 weeks	1.46 (0.82–2.60)	1.77 (1.08–2.91)*
14.1 weeks or greater	0.93 (0.41–2.14)	1.17 (0.60–2.29)
**Previous abortion**	0.81 (0.51–1.28)	1.01 (0.69–1.47)
**Previous birth**	0.96 (0.63–1.47)	1.11 (0.77–1.61)
**Baseline decision conflict score** (Low conflict (<25))		
Medium conflict (25–37.5)	1.03 (0.60–1.75)	1.10 (0.70–1.73)
High conflict (>37.5)	1.47 (0.93–2.33)	1.37 (0.92–2.05)
**Distance to nearest CPC** (Less than 5 miles)		
5–24 miles	0.57 (0.37–0.88)*	0.72 (0.50–1.05)
25+ miles	0.59 (0.26–1.33)	0.68 (0.34–1.36)
**Distance to nearest abortion facility** (Less than 5 miles)		
5–24 miles	0.97 (0.58–1.61)	0.85 (0.56–1.31)
25+ miles	1.41 (0.83–2.41)	1.13 (0.71–1.78)

*p<0.05

**p<0.01.

†Other race/ethnicity includes native Hawaiian, Pacific Islander, American Indian, and Alaska Native; Other religion includes Buddhism, Hinduism, Islam, Judaism, and Mormonism.

*Notes*: “Not sure/other” health insurance and missing gestation categories not shown. “Possible misclassification” is inclusive of confirmed CPC, missing name of CPC, and could not be determined.

**Fig 3:**
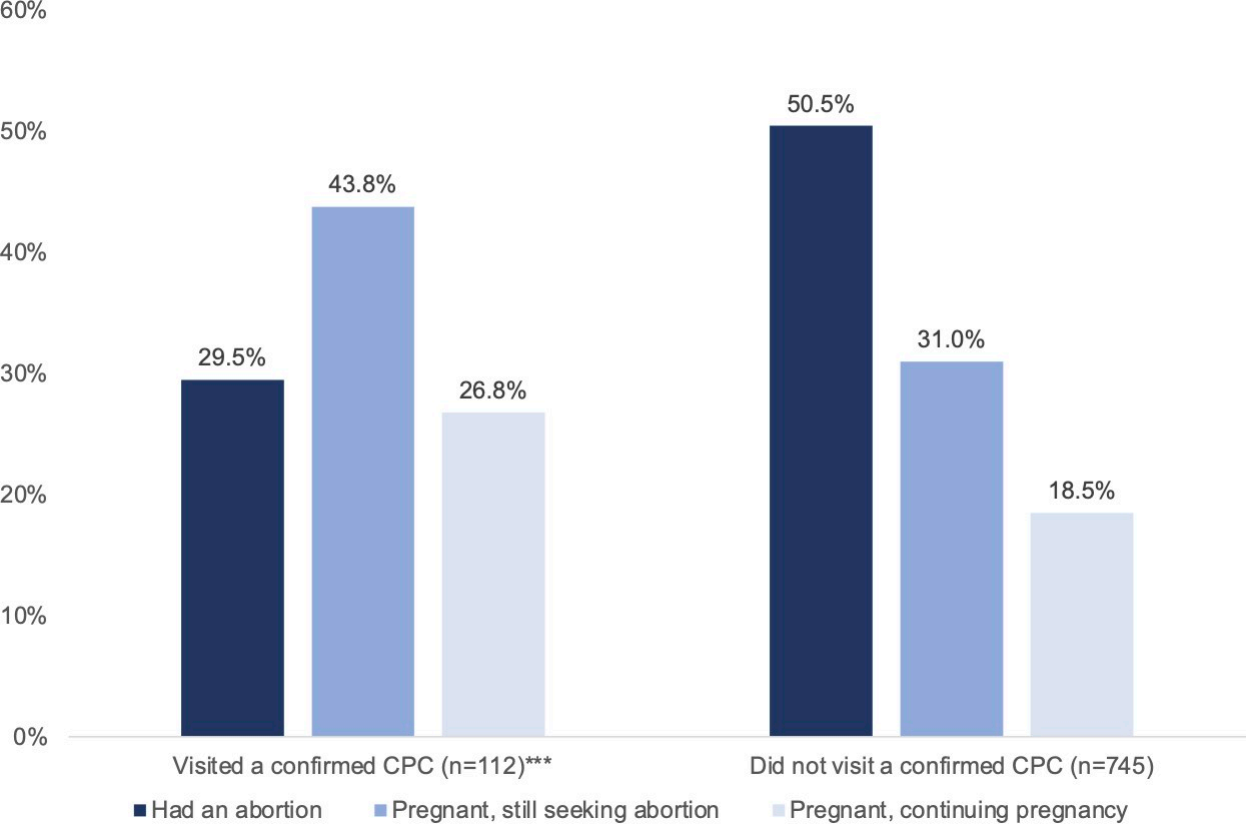
Pregnancy and abortion outcomes at follow-up survey, by confirmed CPC visit. ***p<0.001.

In bivariate analyses, significantly fewer respondents who had visited a confirmed CPC had had an abortion by follow-up (29.5% compared to 50.5% among those who had not visited a CPC) (Fig 3). The largest proportion of respondents who had visited a confirmed CPC were pregnant and still seeking an abortion at follow-up (43.8%).

In the multinomial analysis, respondents who reported visiting a confirmed CPC had over twice the odds of being pregnant and still seeking abortion (rather than having had an abortion) at follow-up (aOR = 2.26, 95% CI: 1.37–3.73), compared to respondents who did not visit a confirmed CPC (Table 3). Similarly, those who visited a confirmed CPC had significantly higher odds of still being pregnant and planning to continue the pregnancy (rather than having had an abortion) at follow-up (compared to those with no reported CPC visit) (aOR: 2.35, 95% CI: 1.33–4.15). Both the sensitivity analyses (with the possible misclassified respondents and propensity score-adjusted) yielded similar results.

**Table 3:** Adjusted odds ratios from mixed effects logistic regression models assessing associations between visiting a confirmed CPC and being pregnant and seeking abortion or continuing pregnancy compared to having had an abortion at 4-week follow-up (N = 857).

	Pregnant, still seeking abortion	Pregnant, continuing pregnancy
**Model 1: Main adjusted model**		
Visited a confirmed CPC	2.26 (1.37–3.73)**	2.35 (1.33–4.15)**
Did not visit a confirmed CPC (ref)	--	--
**Model 2: Main adjusted model including potential misclassified respondents**		
Visited a confirmed CPC	1.56 (1.02–2.38)*	2.13 (1.32–3.42)**
Did not visit a confirmed CPC (ref)	--	--
**Model 3: Propensity-score adjusted model**		
Visited a confirmed CPC	2.19 (1.25–3.84)**	2.64 (1.33–5.25)**
Did not visit a confirmed CPC (ref)	--	--

*p<0.05

**p<0.01.

*Notes*: Models 1 and 2 adjusted for age, race/ethnicity, education, difficulty meeting basic needs, health insurance, religion, religiosity, gestation of pregnancy, previous abortion, previous abortion, baseline decision conflict score, distance to nearest CPC, and distance to nearest abortion facility. ref = reference group.

## Discussion

In this study of pregnant people searching online for abortion information, 13.1% reported visiting a confirmed CPC. This national estimate is higher than the proportion found in a previous study conducted in a single state [12]. This analysis found that visiting a confirmed CPC was significantly associated with being pregnant and still seeking abortion or planning to continue the pregnancy at follow-up, even when controlling for other baseline factors.

While research has documented efforts by the CPC movement to specifically target Black and low income women [25], in this analysis, race/ethnicity and difficulty meeting basic needs were not associated with visiting a confirmed CPC. In the sensitivity analysis those without health insurance were significantly more likely to visit CPCs in this sample, possibly because CPCs often offer pregnancy tests or ultrasounds for confirmation of pregnancy free of charge [26]. Additionally, the further people lived from CPCs, the less likely they were to report visiting one, suggesting proximity is a factor to CPC visits. In this sample, distance from an abortion facility was not significantly associated with visiting a CPC. However, it is possible that people may have difficulty distinguishing a CPC from an abortion facility [27], or it may be the case that people who live far from an abortion facility do not feel that accessing abortion is possible [28], and in their absence, readjust their pregnancy expectations and seek information or resources from a CPC.

This analysis is one of the first to look quantitatively at the association between visiting a CPC and abortion or pregnancy outcome among a sample of pregnant people *considering* abortion, generally considered the target population for CPCs. Since those who visited a CPC had significantly higher odds of being pregnant and still seeking abortion at follow-up, compared with those who had not visited a CPC, it is possible that the visit to CPCs delayed people seeking abortion. While this study did not ask respondents if they had found the CPC they visited online, people were recruited into the study through an online search for abortion. Journalists have repeatedly documented instances in which abortion-related searches in Google return results that contain links for CPC websites and locations [29–31]. The services that individual CPCs offer vary, and may be made more confusing by a mix of medical information and misinformation on their websites and during in-person visits [1, 32, 33]. Analyses of CPC websites have found that many advertise the availability of basic medical services on their sites, including pregnancy tests and ultrasounds, and also make reference to “pregnancy options” and abortion, even though they do not provide abortion care [1, 33]. Additionally, our findings suggest that people are still not clear on what distinguishes a CPC from a health care or abortion provider, evidenced by the fact that almost 40% of people who initially stated that they had visited a CPC provided the name of an abortion provider or other legitimate medical provider. Therefore, it is possible that people may have sought information or abortion care from a CPC, only to discover that they did not provide abortion, leading them to still be seeking abortion weeks later.

Having visited a CPC was also associated with higher odds of planning to continue the pregnancy, rather than having had an abortion, even when controlling for sociodemographic, decision conflict, and distance characteristics. As discussed further in the Limitations section, this study did not assess at what time point in their pregnancy respondents visited a CPC. Those who visited earlier in their pregnancy may have had different motivations and experiences than those visiting at later gestations. As qualitative studies have explored [34], pregnant people have obtained material resources and emotional support at CPCs, which may have played a role in encouraging them to continue the pregnancy. People at later gestations may also be realizing that abortion is no longer a realistic option for them due to costs constraints and lack of geographic access to facilities that provide abortions after the first trimester and instead, may be seeking information and resources regarding pregnancy or other material goods, such as baby clothes and supplies [34, 35]. Alternately, as noted above, people living far from abortion facilities may have sought out abortion care or referrals from CPCs, and not being linked to abortion care, may see continuing their pregnancy as the only option.

However, it is also possible that information provided at a CPC visit played a role in convincing people to continue their pregnancies. As has been noted from a “secret shopper” study conducted in North Carolina, inaccurate information on abortion and breast cancer, infertility, and mental health problems was provided to people during in-person CPC visits [32]. This false information, as well as abortion stigma reinforced by CPCs, may convince people that abortion is a greater health risk than continuing a pregnancy, even though the risk of death from childbirth is approximately 14 times that of abortion [36].

### Strengths and limitations

Some key strengths of this study are its multi-state sampling design including all 50 states and the District of Columbia, as well as recruitment of respondents while they were still considering their pregnancy or abortion options. In addition, by asking respondents to provide the name and location of the CPC they visited, we were able to confirm existing CPCs, as well as rule out abortion facilities and primary health care providers. The 13% of the sample who visited CPCs is in fact a conservative estimate, given that respondents whose visits to CPCs could not be confirmed were classified as not exposed in the main analyses. Yet even with additional sensitivity analyses, the primary associations persisted.

However, while this study recruited participants from every state, it was not nationally representative of all individuals considering abortion. Access to the internet may have limited the participation of lower-income or more rural respondents, and privacy or safety concerns may have affected baseline participants’ willingness to provide an email address or cell phone number for follow-up. In addition, due to differential loss-to-follow up, the analysis sample was older, with a higher proportion of people who were white and more educated, had less difficulty meeting their basic needs, and were less likely to have had a previous birth than the baseline sample originally recruited. However, losing respondents with less access to monetary and health system resources in follow-up may actually have caused us to underestimate the association between visiting a CPC and pregnancy outcome, making our estimates in this paper conservative. We are not able to comment on how respondents identified the CPC they visited and what information or resources people were seeking compared to what they actually obtained from CPCs and we do not know if people were seeking out CPCs intentionally or mistakenly thought that they there were abortion facilities or other health care providers. Future research should consider asking whether people knew the CPC was not an abortion facility before their visit to quantify the degree to which people are being misled in their search for health care. Finally, we did not ask respondents if they believed that their experience at the CPC directly affected their pregnancy or abortion decision.

## Conclusion

This paper contributes one of the first estimates of the proportion of pregnant people considering abortion who visit CPCs during their pregnancy, which appears to be at least 10% among a sample seeking information on abortion online. Reproductive and maternal health care providers and public health practitioners should seek innovative ways to reach pregnant people seeking information about pregnancy or abortion, including online, to ensure they have access to unbiased information on resources available for free or at low-cost. Research should continue to investigate what services pregnant people considering abortion are seeking from CPCs and whether those needs are being met, or if people are being misled about the care that is available. It is imperative that individuals considering abortion have unbiased information about their pregnancy options so that they can make the best decision for themselves.
